# Urinary Exosomal miRNAs as Non-Invasive Biomarkers Linked to Podocyte Morphometry in CKD

**DOI:** 10.3390/cells15070593

**Published:** 2026-03-26

**Authors:** Tim Lange, Luzia Maron, Stefan Simm, Silvia Ribback, Heiko Dunkel, Sabrina von Rheinbaben, Tilman Schmidt, Florian Siegerist, Matthias Nauck, Sabine Ameling, Sören Franzenburg, Christian Scheer, Vedran Drenic, Tim Endlich, Gregor Hoppstock, Uwe Zimmermann, Uwe Völker, Sylvia Stracke, Peter R. Mertens, Nicole Endlich

**Affiliations:** 1 Department of Anatomy and Cell Biology, University Medicine Greifswald, 17487 Greifswald, Germany; 2 Institute of Bioinformatics, University Medicine Greifswald, 17475 Greifswald, Germany; 3 Faculty of Applied Natural Sciences and Health, Coburg University of Applied Sciences and Art, 96450 Coburg, Germany; 4 Institute for Pathology, University Medicine Greifswald, 17475 Greifswald, Germany; 5 Department of Internal Medicine C, University Medicine Greifswald, 17475 Greifswald, Germany; 6 Nephrology, Department of Internal Medicine A, University Medicine Greifswald, 17475 Greifswald, Germany; 7 Neonatology and Pediatric Intensive Care Medicine, Department of Pediatrics and Adolescent Medicine, University Medicine Greifswald, 17475 Greifswald, Germany; 8 German Center for Child and Adolescent Health (DZKJ), Partner Site Greifswald/Rostock, 17475 Greifswald, Germany; 9 Institute of Clinical Chemistry and Laboratory Medicine, University Medicine Greifswald, 17475 Greifswald, Germany; 10 German Centre for Cardiovascular Research (DZHK), Partner Site Greifswald, University Medicine Greifswald, 17475 Greifswald, Germany; 11 Interfaculty Institute for Genetics and Functional Genomics, University Medicine Greifswald, 17475 Greifswald, Germany; 12 Institute of Clinical Molecular Biology, Christian-Albrechts-University of Kiel, 24118 Kiel, Germany; 13 Department of Anaesthesiology and Intensive Care, University Medicine Greifswald, 17475 Greifswald, Germany; 14 NIPOKA GmbH, Center of High-End Imaging, 17489 Greifswald, Germany; 15 University Clinic for Nephrology and Hypertension, Diabetology and Endocrinology, Otto-von-Guericke University Magdeburg, 39106 Magdeburg, Germany; 16 Department of Urology, University Medicine Greifswald, 17475 Greifswald, Germany

**Keywords:** CKD, podocyte, miRNA, biomarker, exosome, urine, super-resolution microscopy

## Abstract

Chronic kidney disease (CKD) is a major global health burden leading to a loss of kidney function via podocyte damage, a non-regenerative renal cell type. Early detection of podocyte injury is crucial but remains limited, highlighting the need for non-invasive biomarkers. Therefore, we analysed urinary exosomal microRNAs (miRNAs) in relation to podocyte morphology in biopsies from 65 CKD patients, including focal segmental glomerulosclerosis (FSGS), minimal change disease (MCD) and healthy controls. Global profiling distinguished CKD patients from controls, with miR-606 consistently upregulated and miR-431 downregulated. In podocytopathies, MCD displayed a predominantly suppressed miRNA profile, with miR-141, miR-429, and miR-660 as key candidates, whereas FSGS exhibited elevated miR-181c, miR-3610, miR-663b, miR-4651, and miR-429. Super-resolution morphometry revealed diffuse foot process effacement in MCD and heterogeneous, focally disrupted architecture in FSGS, providing a structural context for the molecular findings. Regression analyses linked these miRNAs to filtration slit density and length, proteinuria, and 25-Hydroxy-vitamin-D3 levels, integrating molecular, structural, and clinical readouts. These results define a coherent miRNA signature of podocyte injury that distinguishes CKD entities and correlates molecular changes with disease severity. Combining urinary exosomal miRNAs with morphometric analysis facilitates early, non-invasive identification of podocyte damage, enabling earlier therapeutic intervention in podocytopathies.

## 1. Introduction

Chronic kidney disease (CKD) affects about 10% of the world’s population and is expected to be among the top five causes of years of life lost by 2040 [1,2]. Mainly attributable to arterial hypertension and diabetes, this terminal disease ultimately progresses to end-stage kidney disease (ESKD) requiring renal replacement therapy like kidney transplantation or hemodialysis, as no causal therapies are available. In order to decelerate disease progression or ESKD and the associated loss of quality of life, diagnosis in the early stages of the disease is desirable. However, current parameters in clinical diagnostics are limited in this respect.

ESKD is predominantly caused by glomerulopathies, with its pathogenesis essentially determined by effacement and loss of podocytes, a specialized post-mitotic cell type that is crucial for the integrity of the glomerular filtration barrier [3]. Podocyte function and, therefore, proper kidney function depend considerably on miRNAs [4]. These small, non-coding nucleic acids, which act as posttranscriptional regulatory elements through mRNA decay or translational repression, have been shown to be differentially expressed in certain entities of CKD [5]. As a result, miRNAs are investigated not only for their involvement in the pathogenesis of kidney diseases but also for their potential as biomarkers.

Beyond their expression in tissue samples, miRNAs can further be found in exosomes. These endosome-derived extracellular vesicles are secreted by all cell types under physiological and pathological conditions and contain cellular proteins, nucleic acids, lipids and other metabolites [6]. Among other body fluids, exosomes have been detected in urine and received great attention as a diagnostic tool, given that sampling is non-invasive and easily accessible [7]. As a result of their biogenesis, exosomes possess a lipid bilayer membrane that protects encapsulated miRNAs from degradation through nucleases. Along with their stability across different storage conditions, these properties highlight their great utility in diagnostic applications [8].

Several previous studies have focused on differential urinary exosomal miRNA expression among CKD patients. Compared to the control group, Lv and coworkers showed that miR-29c levels were significantly decreased in CKD patients, and this decrease correlated with a decline in glomerular filtration rate (GFR) and degree of renal fibrosis [9]. Our group identified miR-21 to be up-regulated in urinary exosomes of patients with CKD and being inversely correlated with GFR [10].

Although these studies provide initial insights into the differential expression of pre-selected exosomal miRNA candidates, larger-scale analysis and more comprehensive data are required to evaluate a broader spectrum of miRNAs dysregulated in kidney diseases. Furthermore, little is known about how miRNA expression patterns may vary across different CKD entities. Importantly, previous studies did not integrate miRNA expression data with high-precision biopsy diagnostics, which is crucial to establishing direct morphological correlations with podocyte injury. Such correlations are essential to link molecular signatures to structural alterations in the glomerular filtration barrier and thereby to better understand disease mechanisms and progression.

The present study was conducted to investigate expression levels of different miRNAs in urinary exosomes of CKD patients with regard to distinct disease subtypes, specifically focusing on focal glomerular sclerosis (FSGS) and minimal change disease (MCD). To combine miRNA expression data with the status of kidney damage, we performed morphological and quantitative analysis of the podocyte filtration slit by the podocyte exact morphological measurement procedure (PEMP).

## 2. Methods

### 2.1. Patient and Control Samples

Urine samples and kidney biopsies were collected from patients by the Department of Internal Medicine A and the healthy controls were collected by the Department of Urology at University Medicine Greifswald, as well as by the University Clinic for Nephrology, Hypertension, Diabetology, and Endocrinology at Otto-von-Guericke University Magdeburg. All participants provided written informed consent. The study was approved by the local ethics committee of University Medicine Greifswald and the Otto-von-Guericke University Magdeburg [#BB 166/19(a), 74/09)] and conducted in accordance with the Declaration of Helsinki.

### 2.2. Immunostaining and 3D-SIM

After deparaffinization and rehydration, kidney sections (3–4 µm) were boiled in Tris-EDTA buffer (10 mM Tris, 1 mM EDTA, pH 9) in a pressure cooker for 5 min, followed by blocking in 1% FBS, 1% BSA, 0.1% fish gelatin, and 1% normal goat serum for 45 min. The following primary antibodies were incubated overnight at 4 °C: polyclonal rabbit anti-podocin 1:150 (IBL International, Hamburg, Germany) and monoclonal mouse anti-synaptopodin 1:75 (Progen, Heidelberg, Germany). After washing three times in PBS, secondary antibodies were incubated for 1 h at room temperature: alpaca anti-rabbit Alexa Fluor 488-conjugated VHH antibodies 1:450 (ChromoTek, Planegg, Germany) and alpaca anti-mouse Alexa Fluor 568-conjugated VHH antibodies 1:600 (ChromoTek, Planegg, Germany). DAPI (1:100) was added to the slides for 5 min, followed by 3× washing step with 1× PBS, and 1× ultrapure water and mounted in Vectashield Vibrance (Vector Laboratories, Newark, CA, USA) using high-precision coverslips (Paul Marienfeld, Lauda-Königshofen, Germany). Whole-slide images were first captured by widefield microscopy, followed by 3D-SIM using an N-SIM super-resolution microscope (Nikon, Tokyo, Japan). To account for the 3-dimensional architecture of the filtration slit, 21 z-plane images of all areas of the glomeruli were taken, and a maximum intensity projection (MIP) was performed and stitched together. 3D-SIM images were reconstructed using NIS-Elements AR software 6.10 (Nikon, Tokyo, Japan). Digital post-processing and PEMP were performed using FIJI combined with a custom-built macro as described previously [11]. The algorithm determined the total filtration slit length based on Podocin staining and the total podocyte foot process area based on Synaptopodin staining. The filtration slit density (FSD) was then calculated from the ratio of filtration slit length (FSL) over the podocyte area up to 15 glomeruli per sample.

### 2.3. Urine Processing and Exosomal RNA Isolation

Urine samples were stored at −80 °C and thawed overnight at 4 °C. To remove cells and debris, samples were transferred to Falcon tubes and centrifuged for 3 min at 2000× *g*, followed by 15 min at 2500× *g*. Exosome preparation and miRNA isolation were performed as previously described while omitting the use of RiboShredder™ RNase Blend (Epicentre, Madison, WI, USA) [12]. Eluted RNA was stored at −80 °C.

### 2.4. Small RNA Sequencing

NGS analyses were carried out at the Competence Centre for Genomic Analysis (Kiel). After quality control checkup, small RNA libraries were prepared using the NEXTFLEX Small RNA-Seq Kit v4 (PerkinElmer, Waltham, MA, USA) according to the manufacturer’s protocol. Sequencing was performed on an Illumina NovaSeq 6000 platform (Illumina Inc., San Diego, CA, USA) using an S2 flow cell with a paired-end 2 × 50 bp configuration.

### 2.5. Mapping and Counting

Total smallRNA-seq data from the two cohorts of Greifswald and Magdeburg were mapped to the Ensembl human genome version GRCh38.p14 using NextGenMap (https://github.com/Cibiv/NextGenMap (accessed on 2 October 2025), version 0.5.0 [13]) with the settings -b, --no-unal, and --very-sensitive. After mapping, expression values for the total smallRNA-seq data were determined by counting using featureCounts [14]. For the total smallRNA-seq data, the complete Ensembl annotation of the human genome (version GRCM39.113.chr.gtf) was used for counting with the gene and gene_id, while for the counting of miRNA we used the same gtf but miRNA and gene_id to filter only miRNAs. For the counting of the total smallRNA-seq data, the settings -M were used while all other parameters were kept at their default settings. Counting tables from the total smallRNA and miRNA only were processed in parallel, unless stated otherwise. All analyses were performed using the programming language R 4.5.1 [15]. The counts for all case samples from the two recruiting sites (University Medicine Greifswald, HGW and Otto-von-Guericke University Magdeburg, MD) were merged into a single table. The control samples were sequenced twice, once at each sequencing from the corresponding recruiting site. The counts from the corresponding samples from the two sites were combined by summation.

### 2.6. Batch Effect Correction and Differential Expression Analysis

To account for batch effects between the two sequencing sites, we performed batch effect correction for all case samples, treating site HGW as one batch and site MD as the other. ComBat-seq [16] was used to detect and correct batch effects. The merged control samples were added to the batch effect-corrected case samples and normalized using the standard DESeq2 procedure [17]. The normalized counts were used as input for differential expression analysis with all case samples pooled and compared to the control samples. As we were interested in specific miRNA and RNA biomarkers for kidney diseases, we continued our analysis of the dataset as follows: First, we performed differential expression analysis for each disease individually versus the three control samples to identify specific biomarkers for each disease compared to healthy subjects. Next, we performed differential expression analysis of every combination of diseases to distinguish diseases from each other.

All kidney diseases with <3 samples were dropped from the disease-specific analysis. Furthermore, when only a single sample for a specific diagnosis was available at site HGW, it was dropped. The exact number of samples after each disease for each disease can be found in Table 1. Filtering left 65 disease samples (HGW: 18; MD: 47), opposing 3 control samples.

### 2.7. Unsupervised Machine Learning Approaches: PCA and Clustering

After applying variance-stabilizing transformation (vst) to the counts, principal component analysis (PCA) was performed using the sva R package 4.5.1 [18]. The first principal components were plotted using ggplot2 4.0 [19].

To derive specific miRNA signatures, we combined the Log_2_ fold change (Log_2_FC) of each miRNA (compared to the control samples) for each disease and created a table (rows: miRNAs; columns: diseases). Additionally, we created another table with the same structure by taking the median normalized count value of each miRNA for each disease. These counts were normalized using Z-score normalization per row. Both tables were used as input for clustering using Euclidean distance in Orange (https://orangedatamining.com/, accessed on 2 October 2025) [20]. These cluster maps were analysed for specific miRNAs or groups of miRNAs that could distinguish between different diseases.

### 2.8. Statistical Analysis and Regressions

In accordance with the standard DESeq2 procedure, the Wald test was used to test the significance of the Log_2_FC of each gene for differential expression analysis [17]. Multiple testing correction was performed using Benjamini-Hochberg correction [21]. MiRNAs and all RNAs are considered differentially expressed if the adjusted two-sided *p*-value (*p*-adjust) is lower than 0.05. For the linear univariate and multivariate regression analysis, we used Sigmaplot (version 12.5 and the regression wizard) to perform regression analysis between the FSGS phenotypes and the miRNA expression level.

### 2.9. Analytical Workflow

To ensure a transparent analytical strategy and minimize bias from heterogeneous disease composition, we applied a stepwise workflow for the analysis of urinary exosomal small RNA sequencing data. Raw reads from both cohorts were mapped to the human reference genome (GRCh38) and quantified as described above. After merging count tables from the two sequencing centers, batch effects related to the sequencing site were corrected using ComBat-seq, followed by variance-stabilizing normalization in DESeq2 package in R 4.5.1.

Global expression patterns were first explored using unsupervised approaches, including principal component analysis (PCA) and hierarchical clustering of variance-stabilized counts, performed at the level of individual samples so that each sample contributed equally regardless of subgroup size.

Differential expression analyses were then conducted hierarchically: (i) CKD samples versus healthy controls to identify miRNAs broadly associated with kidney disease, (ii) entity-specific comparisons of individual CKD subtypes versus controls, and (iii) pairwise comparisons between CKD subtypes to identify miRNAs distinguishing closely related podocytopathies, particularly FSGS and MCD. Entities with fewer than three samples were excluded from subtype-specific analyses. Differential expression testing was performed using the Wald test in DESeq2 with Benjamini–Hochberg adjustment, and miRNAs with adjusted *p* < 0.05 were considered significant.

This strategy enabled the separation of global CKD-associated miRNA patterns from entity-specific signatures while minimizing the influence of uneven subgroup sizes.

## 3. Results

### 3.1. Patient Characteristics

A total of 68 individuals were included, comprising 65 CKD patients and three healthy controls. CKD patients had a mean age of 56 (±14) years, and 46.2% were female. CKD patients had a mean age of 56 ± 14 years, with 46.2% being female. The CKD cohort included seven distinct disease entities, including 22 patients with diabetic nephropathy, 12 with FSGS, and 5 with MCD. Further details on the distribution of the remaining subgroups are provided in Table 2. The mean GFR in the CKD group was 38.8 (±28.1) mL/min. Data on 55 patients were available to determine the urinary albumin-to-creatinine ratio (ACR), resulting in a mean of 620.3 (±827.1) mg/g. The mean urinary protein-to-creatinine ratio (PCR) for these patients was 966.6 (±1422.4) mg/g. Data on ACR and PCR were not collected for healthy controls. Reference ranges for albuminuria and proteinuria according to KDIGO are: Normal: ACR < 30 mg/g, PCR < 150 mg/g; Moderate: ACR 30–300 mg/g, PCR 150–500 mg/g; Severe: ACR > 300 mg/g, PCR > 500 mg/g [22].

### 3.2. Global Separation of CKD and Controls by Urinary Exosomal miRNAs

**Figure 1:**
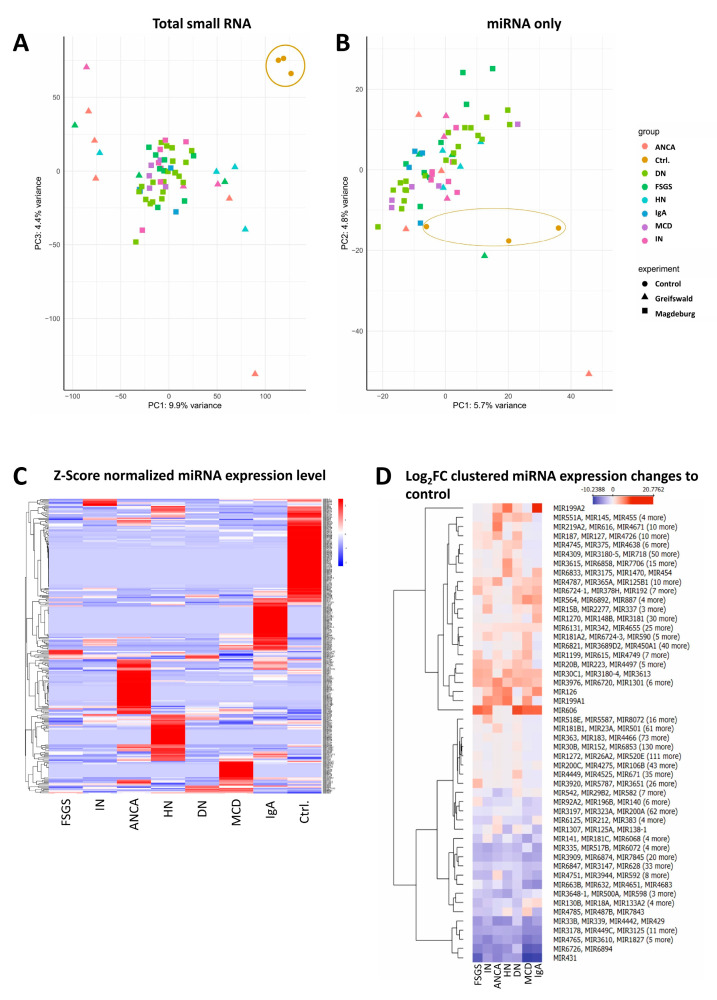
Global separation of CKD and controls by urinary exosomal microRNAs. (**A**) Principal component analysis (PCA) of all RNA subclasses clearly separated CKD samples from healthy controls, with partial sub-clustering of disease entities but no cohort-specific clustering. (**A**,**B**) Controls are shown as round points, Greifswald samples as triangles, Magdeburg samples as squares; miRNA entities are color-coded consistently. (**B**) PCA of miRNAs alone also separated CKD samples from controls, though less pronounced than for all RNA subclasses. (**C**) Z-score clustering revealed distinct miRNA expression patterns specific to control samples and CKD subgroups. (**D**) Differential expression analysis highlighted disease-associated miRNA signatures, with miR-606, miR-3177, and miR-22 among the most significantly upregulated, and miR-431, miR-520c, and miR-6728 (miR-6728 included in grouped category) among the most downregulated. ANCA = Anti-neutrophil cytoplasmic (ANCA)-associated vasculitis, DN = diabetic nephropathy, FSGS = focal segmental glomerulosclerosis, HN = hypertensive nephropathy, MCD = minimal change disease, IN = no blood pressure damage + interstitial nephritis, IgA = IgA nephritis + no blood pressure damage.

Unsupervised analysis of normalized urinary exosomal miRNA counts showed a clear separation between CKD patients and healthy controls. Principal component analysis (PCA) of all small RNA subclasses (Figure 1A) showed clear clustering of disease samples away from controls, with some sub-clustering of disease entities. No cohort-specific clusters were observed. PCA of miRNAs only (Figure 1B) also revealed a distinct separation of control samples from CKD but to a lower extent than all RNA subclasses. Likewise, we could not detect any clusters separating the different cohorts. As revealed by Z-score clustering, we could identify clear miRNA clusters specific for the control group and the different CKD entities (Figure 1C). To further quantify these differences, we performed differential expression analysis using Log_2_FC, thereby pinpointing candidate genes with the strongest regulation between conditions (Figure 1D). Thereby, we could confirm the disease-specific miRNA expression patterns. Amongst several others, we could identify miR-606 (Log_2_FC = 7.23, *p* = 0.0003), miR-3177 (Log_2_FC = 6.21, *p* = 0.02) and miR-22 (Log_2_FC = 5.36, *p* = 0.01) as the most upregulated significant candidates overall. Vice versa, we could identify miR-431 (Log_2_FC = −5.85, *p* = 0.05), miR-520c (Log_2_FC = −5.24, *p* = 0.05) and miR-6728 (Log_2_FC = −3.84, *p* = 0.002) as the most downregulated significant candidates overall. MiR-606 was strongly upregulated in FSGS, interstitial nephritis without blood pressure damage (nBP + IN), diabetic nephropathy, IgA nephropathy, and MCD, but not in anti-neutrophil cytoplasmic (ANCA)-associated vasculitis or hypertensive nephropathy. Interestingly, specifically miR-431 was downregulated to a larger extent in FSGS, MCD and in IgA.

### 3.3. Specific miRNA Expression in FSGS and MCD

**Figure 2:**
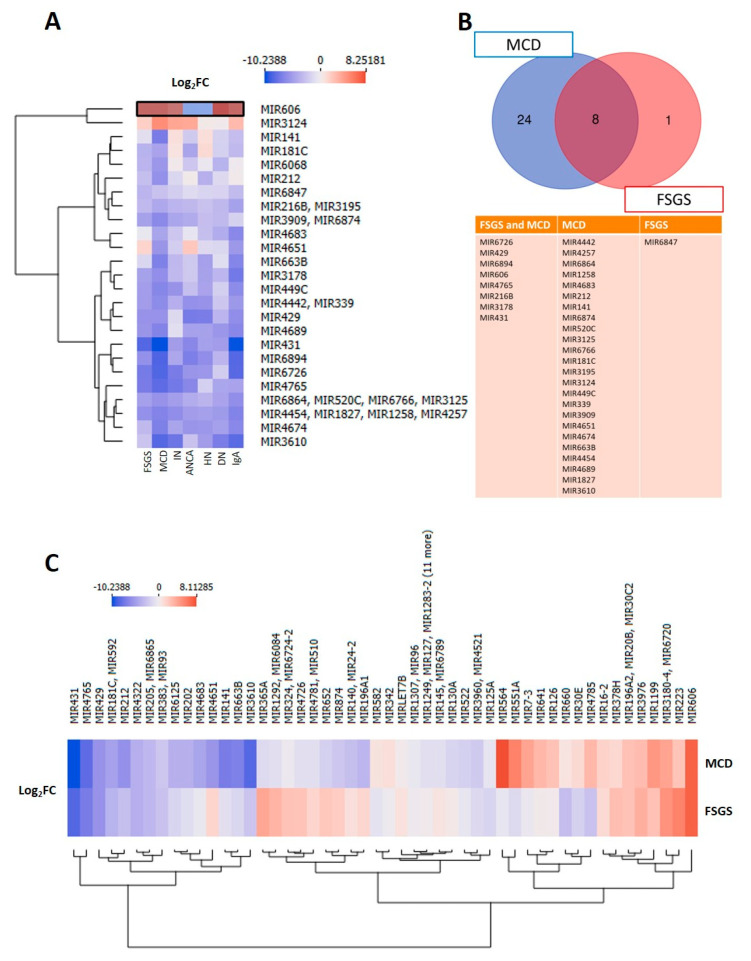
Differentially expressed microRNAs in MCD and FSGS. (**A**) Differential expression analysis of miRNAs uniquely deregulated in FSGS and MCD relative to healthy controls identified miR-606 (black box) as the most upregulated and miR-431 as the most downregulated candidate. (**B**) Eight miRNAs were deregulated in both FSGS and MCD, 24 miRNAs exclusively in MCD, and one miRNA exclusively in FSGS. (**C**) Differential expression analysis between FSGS and MCD revealed a predominantly downregulated miRNA signature in MCD, including miR-141, miR-4651, and miR-3610, whereas miR-660 was the only significantly upregulated candidate in MCD. ANCA = Anti-neutrophil cytoplasmic (ANCA)-associated vasculitis, DN = diabetic nephropathy, FSGS = focal segmental glomerulosclerosis, HN = hypertensive nephropathy, MCD = minimal change disease, IN = no blood pressure damage + interstitial nephritis, IgA = IgA nephritis + no blood pressure damage.

Because MCD and FSGS are primarily driven by podocyte injury, unlike other CKD subtypes, we specifically focused on these two entities. By selecting differentially expressed miRNAs (DEMs) in FSGS and MCD cases relative to healthy controls, we identified 33 candidate miRNAs. From them, miR-606 showed the strongest upregulation and miR-431 the most prominent downregulation (Figure 2A). From all 33 DEMs, only eight miRNAs showed significant differences in FSGS and MCD compared to the control probands, whereas 24 DEMs were specific for MCD, and only one DEM (miR-6847) was specific for FSGS (Figure 2B). After identifying differentially expressed miRNAs (DEMs) in FSGS and MCD patients compared to controls, we subsequently analysed miRNAs that were differentially expressed between FSGS and MCD in order to distinguish the two entities. Overall, 75 DEMs could be identified (Figure 2C), of which 11 were also DEMs in comparison to the healthy group (Figure 2C). Several miRNAs were markedly downregulated in MCD compared to FSGS, including miR-141 (Log_2_FC = −6.46, *p* = 9.1 × 10^−5^), miR-4651 (Log_2_FC = −7.44, *p* = 0.006), and miR-3610 (Log_2_FC = −6.28, *p* = 0.013). Additional downregulated candidates included miR-4683, miR-874, miR-196A1, miR-140, miR-365A, miR-663B, miR-24-2, and miR-324, all with Log_2_FC −2.85 and −5.55 and reaching nominal significance. In contrast, miR-660 was the only miRNA found to be significantly upregulated in MCD specifically compared to FSGS (Log_2_FC = 3.61, *p* = 0.018). These findings highlight a predominantly downregulated miRNA signature in MCD relative to FSGS, with miR-660 emerging as a potential exception (Figure 2C).

### 3.4. Podocyte Morphometry Shows Typical Histopathological Features of CKD Entities

**Figure 3:**
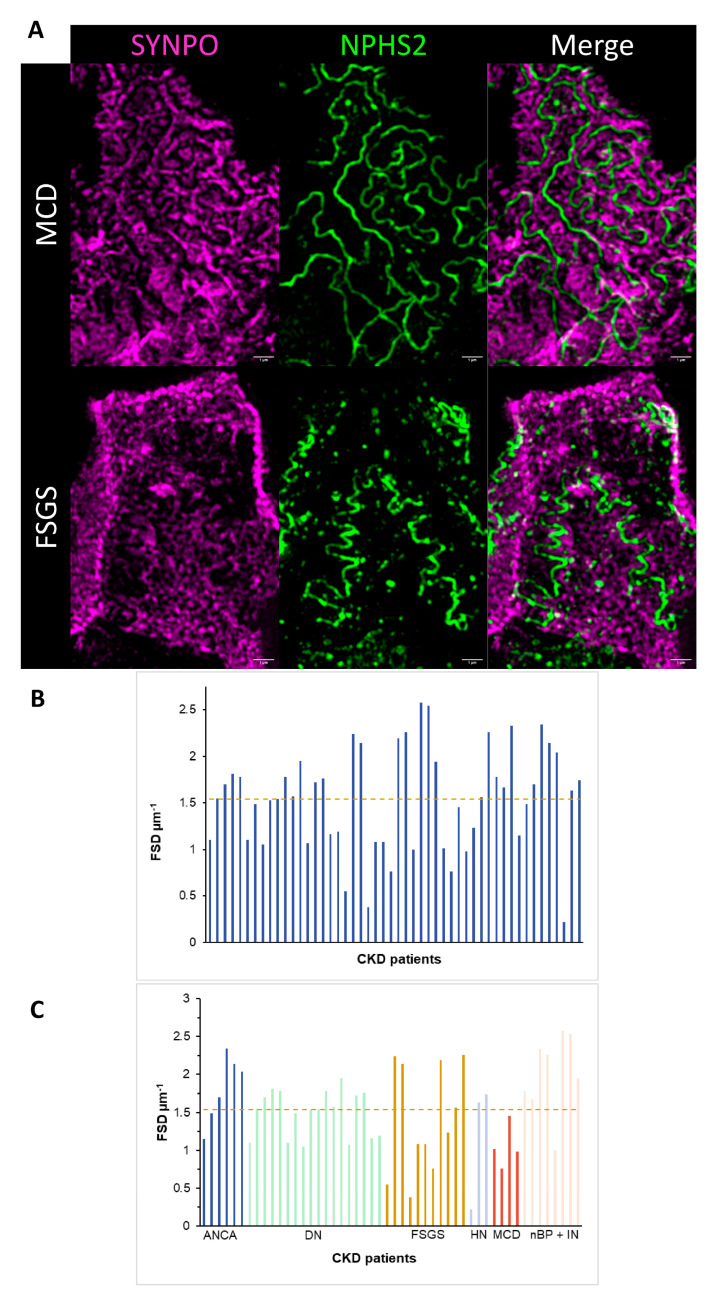
Representative structured illumination microscopy images of glomerular sections from patients with minimal change disease (MCD) and focal segmental glomerulosclerosis (FSGS). (**A**) Formalin-fixed, paraffin-embedded kidney sections (3–4 µm) were stained for Synaptopodin (SYNPO, magenta) and podocin (NPHS2, green) and analysed using the podocyte exact morphological measurement procedure (PEMP). Merged images show differences in podocyte structure and marker distribution between MCD and FSGS. Scale bars: 1 µm. (**B**) Quantitative analysis of foot process slit density (FSD) across all CKD patients, shown as individual values, and (**C**) grouped by disease subtypes. ANCA = Anti-neutrophil cytoplasmic (ANCA)-associated vasculitis, DN = diabetic nephropathy, FSGS = focal segmental glomerulosclerosis, HN = hypertensive nephropathy, MCD = minimal change disease, nBP + IN = no blood pressure damage + interstitial nephritis. Scattered line = mean FSD. Yellow dashed line show mean FSD/FSL.

To analyse podocyte foot process integrity in the CKD biopsies corresponding to the urine samples, we applied the podocyte exact measurement procedure (PEMP), a structured illumination microscopy (SIM)-based technique enabling super-resolved assessment of slit membrane and foot process morphology. PEMP revealed podocyte effacement and reduced podocin expression in almost all disease entities (Figure 3A–C), consistent with their characteristic histopathological features.

Quantitative PEMP measurements showed a mean filtration slit length (FSL) of 5930.04 ± 4394.6 and a mean filtration slit density (FSD) of 1.54 ± 0.55 across all patients (Figure 3B), highlighting substantial heterogeneity of podocyte remodelling within the cohort. Importantly, SIM-based imaging and quantitative slit analysis clearly distinguished MCD from FSGS (Figure 3A). MCD samples exhibited continuous SYNPO and NPHS2 labelling patterns, reflecting a more uniformly altered but structurally coherent podocyte foot process morphology. In contrast, secondary FSGS biopsies showed irregular, discontinuous, and structurally disrupted staining, consistent with the focal nature of the disease.

Subgroup analysis of FSD further supported these disease-specific differences. While MCD showed the lowest FSD values (1.05 ± 0.29), reflecting diffuse foot process effacement, FSGS had a higher mean FSD (1.41 ± 0.71) with greater variability, indicating the coexistence of intact and severely damaged regions characteristic of this entity. Hypertensive nephropathy showed similarly broad variability (1.20 ± 0.85), suggesting inconsistent structural damage across glomeruli. Intermediate FSD values were found in diabetic nephropathy (1.49 ± 0.30), whereas ANCA-associated glomerulonephritis demonstrated comparatively preserved slit density (1.81 ± 0.45). The highest FSD values occurred in the nBP + IN subgroup (2.01 ± 0.53), indicating a relatively intact podocyte morphology (Figure 3C).

Overall, these findings reveal pronounced differences in podocyte structure across CKD entities. While FSGS and hypertensive nephropathy show the most heterogeneous and severely disrupted morphology, ANCA and nBP + IN retain comparatively preserved slit membranes. 3D-SIM-based PEMP thus provides a high-resolution and sensitive means to discriminate disease-specific patterns of podocyte injury that are not fully captured by conventional qualitative assessment.

### 3.5. Correlations Show Potential of Podocyte Morphometry to Improve Clinical Parameter Set for Kidney Disease Diagnostics

As clinical parameters for kidney diseases like glomerular filtration rate (GFR), 25-Hydroxy-vitamin-D3 (VitD), urinary albumin-to-creatinine ratio (ACR), and urinary protein-to-creatinine ratio (PCR) are sometimes hard to correlate with disease severity, we wanted to test their correlation to super-resolution morphometrical podocyte analyses. Here, we mainly focused on FSGS due to the low case number within the MCD cohort.

**Figure 4:**
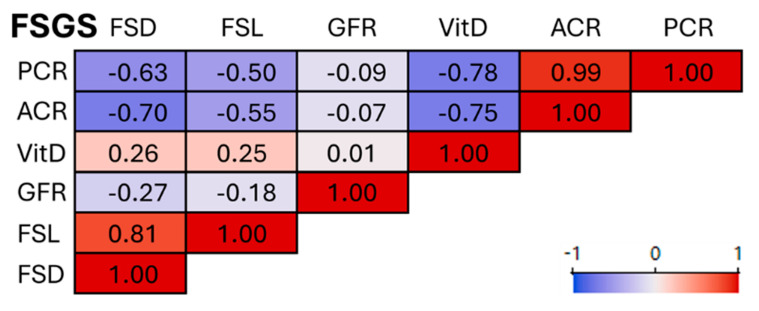
Correlation of podocyte morphometry with clinical parameters in focal segmental glomerulosclerosis (FSGS). In FSGS, FSD and FSL were inversely correlated with proteinuria (ACR, PCR), indicating that reduced podocyte structural integrity is associated with higher proteinuria. 25-Hydroxy-vitamin D3 (VitD) levels correlated negatively with ACR and PCR, and weakly positively with podocyte morphometric parameters, while GFR showed no strong associations. FSGS = focal segmental glomerulosclerosis, FSD = filtration slit density, FSL = filtration slit length, GFR = glomerular filtration rate, VitD = 25-Hydroxy-vitamin-D3, ACR = urinary albumin-to-creatinine ratio, PCR = urinary protein-to-creatinine ratio.

In the FSGS cohort, correlation analysis revealed clear associations between clinical parameters. FSD and FSL were strongly positively correlated (r = 0.81) and inversely correlated with albumin-to-creatinine ratio (ACR; r = −0.70 and −0.55) and protein-to-creatinine ratio (PCR; r = −0.63 and −0.50), suggesting that reduced podocyte structural integrity is associated with increased proteinuria. Furthermore, VitD levels correlated negatively with ACR (r = −0.75) and PCR (r = −0.78), while showing a weak positive correlation with FSD and FSL. Glomerular filtration rate (GFR) did not show strong associations with the other parameters. Overall, these findings indicate that podocyte morphology and VitD status are closely linked to proteinuria severity in FSGS patients (Figure 4).

### 3.6. Regression Analysis Suggests miRNA Candidates as Targets for MCD and FSGS

**Figure 5:**
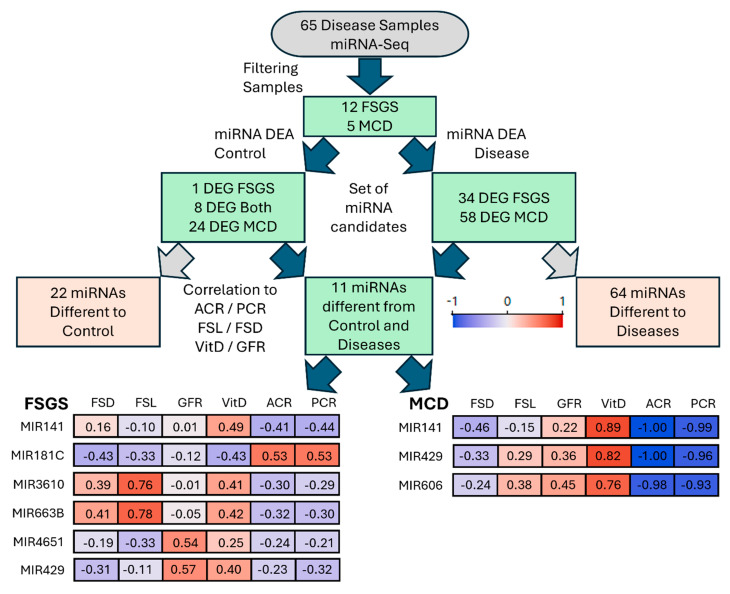
Flow diagram of miRNA candidate selection and correlations with podocyte morphometry and clinical parameters in FSGS and MCD. Differential expression analysis comparing controls versus all disease samples, as well as pairwise comparisons between disease entities, identified 11 candidate miRNAs for FSGS and MCD: miR-141, miR-181c, miR-3610, miR-663b, miR-4651, and miR-429 for FSGS, and miR-141, miR-429, and miR-606 for MCD. The accompanying power heatmaps illustrate the correlations between miRNA expression and phenotypic metadata for FSGS (**left**) and MCD (**right**), including FSL, FSD, urinary ACR, PCR, VitD, and GFR. Spearman correlation was used for all analyses. These miRNAs exhibited distinct association patterns with podocyte morphology, proteinuria, GFR, and VitD levels. MCD = minimal change disease, FSGS = focal segmental glomerulosclerosis, DEA = differential expression analysis, DEG = differentially expressed genes.

After sorting miRNA expression data according to differences from controls and diseases, respectively, a final set of miRNAs specific for FSGS and MCD was obtained (Figure 5). Regarding FSGS, we identified miR-141, miR-181c, miR-3610, miR-663b, miR-4651 and miR-429 as a specific miRNA pattern in the urine samples. Interestingly, we could observe correlations with clinical parameters and podocyte morphometry. MiR-181c negatively correlated with FSD, FSL and VitD; however, it correlated positively with ACR and PCR. Conversely, miR-3610 and miR-663b exhibited a positive correlation with FSD, FSL and VitD, yet they were negatively correlated with ACR and PCR. MiR-141 showed similar effects. Furthermore, miR-4651 and miR-429 negatively correlated with FSD, FSL, ACR and PCR but positively with GFR.

In MCD, all three candidates—miR-141, miR-429 and miR-606—showed a substantial negative correlation with ACR and PCR. The same marker abundance had a strong positive correlation with VitD. Additionally, we could observe a slight negative correlation with FSD and a moderate positive correlation with eGFR. Taken together, these results indicate that disease-specific miRNA expression patterns may be associated with clinical parameters and podocyte morphometry in FSGS and MCD.

## 4. Discussion

In this study, we demonstrate that urinary exosomal miRNA profiles separate CKD patients from healthy controls and display entity-specific patterns. The combination of control comparisons, direct disease-to-disease analyses, unsupervised clustering, correlation analyses, and differential expression testing based on normalized, batch-corrected, and variance-stabilized counts provides independent evidence for both shared and distinct miRNA signatures across CKD entities, with several candidates emerging as strongly regulated. Among them, miR-606 and miR-431 consistently appeared as highly up- and downregulated species, respectively, across multiple disease groups, including FSGS and MCD. The robustness of these results, despite the small number of controls and the combination of two independent cohorts, supports the potential of urinary exosomal miRNAs as reliable disease-related biomarkers [23]. Notably, miRNA quantification and assay validation in several recent studies have been successfully performed with n = 3 biological replicates, indicating that such sample sizes are commonly used and considered appropriate for exploratory small-RNA sequencing analyses [24,25]. Although the control group was older than the CKD cohort, prior evidence suggests that urinary exosomal miRNA expression, particularly podocyte-associated miRNAs, remains largely stable across age [12,26,27], indicating that the observed CKD-specific and podocyte-related miRNA alterations are unlikely to be substantially confounded by age differences, though validation in larger, age-matched cohorts is warranted.

To date, a limited number of studies have investigated the differentiation between FSGS and MCD by using miRNA expression patterns. MiRNA profiling has only been conducted in serum so far [28], whereas studies on urinary exosomal miRNAs have addressed single miRNAs like miR-193a [29]. Given that increased levels of miR-193a were observed in FSGS but not in MCD and considering its association with podocyte dedifferentiation and disease progression, the enrichment of this urinary exosomal miRNA has been proposed as a potential diagnostic marker. Focusing on these two podocytopathies, we identified a set of urinary exosomal miRNAs that were not only deregulated compared to controls but also allowed discrimination between the two entities themselves. Specifically, MCD was characterized by a predominantly suppressive miRNA signature compared to FSGS, with candidates such as miR-141 [23] and miR-429 markedly downregulated. MiR-141 expression has been shown to be decreased in isolated rat glomeruli after TGFß1 stimulation, leading to epithelial-to-mesenchymal transition (EMT) [30]. Conversely, miR-660 emerged as the only miRNA in our data and was significantly upregulated in MCD compared to FSGS. To date, miR-660 has not been specifically linked to podocyte biology or MCD. In other cellular contexts, however, miR-660 has been implicated in the regulation of proliferation and apoptosis, particularly in cancer biology [31]. The selective upregulation of miR-660 in MCD compared to FSGS observed in our cohort therefore represents a novel finding and may reflect differential regulatory responses rather than established disease mechanisms, warranting further investigation.

We could observe a specific miRNA pattern for FSGS. MiRNAs that were differently expressed from controls and the other diseases were miR-141, miR-181c, miR-3610, miR-663b, miR-4651 and miR-429. MiR-181c is controversially discussed in the literature. In a puromycin aminonucleoside injury model using cultured murine podocytes, miR-181c was found to be significantly upregulated [32]. In contrast, it is known to inhibit EMT and its downregulation is associated with cyclosporin A-induced kidney injury in rats [33]. These contrary roles are most likely to originate from the different injury models [34]. In an Alport syndrome model using induced pluripotent stem cells miR-4651 was 2-fold upregulated compared to controls [35]. Interestingly, there is no data on miR-3610 in the context of kidney research, but it was amongst the most descriptive miRNAs in a pancreatic cancer study [36]. This also applies to miR-663b, which is known to trigger colorectal cancer by inhibition of TNK1 [37].

Taken together, these distinct patterns provide strong evidence that urinary exosomal miRNAs can serve as biomarkers for detecting podocyte injury and distinguishing clinically overlapping yet pathogenetically distinct glomerular diseases. Given the challenges of distinguishing MCD from FSGS by routine histopathology, such molecular tools have particular clinical relevance.

The choice of FSGS and MCD as focus entities is supported by their shared origin as pure podocytopathies. In these diseases, podocyte injury is the primary driver of pathology, in contrast to other pathologies underlying CKD, where vascular, immune, or interstitial components play dominant roles. Urinary exosomes contain podocyte-specific markers, including podocin and podocalyxin, supporting a substantial contribution from glomerular podocytes to the exosomal miRNA pool in CKD [38,39]. This made it possible to directly integrate urinary miRNA data with podocyte morphometry derived from PEMP, thereby linking molecular readouts to structural podocyte alterations. The PEMP data reveal distinct patterns of structural remodelling between the two diseases and demonstrate that FSD is a sensitive indicator of podocyte integrity, which has already been reported before by our group [11,40,41].

Qualitative imaging already highlighted marked differences between MCD and FSGS. Whereas MCD exhibited smooth and continuous SYNPO and NPHS2 staining along the capillary loops—a pattern consistent with a more uniform, though effaced, podocyte morphology—FSGS displayed strikingly fragmented, irregular, and spatially disrupted staining. These patterns reflect the characteristic focal and segmental loss of podocyte architecture seen in FSGS and illustrate its fundamentally heterogeneous injury pattern [42].

These structural differences are not only relevant at the morphological level but also resonate with clinical observations. Correlation analyses reinforced the value of integrating different data layers. In FSGS, PEMP-derived FSD and FSL values correlated with key clinical parameters, such as proteinuria indices. FSGS patients exhibited a negative correlation of FSD and FSL with PCR and ACR. Since the development of proteinuria is closely related to a proper podocyte morphometry [3,11], our findings in FSGS are fully consistent with these facts. Additionally, VitD levels were positively correlated with ACR and PCR in FSGS. VitD is known to have an impact on cell integrity by orchestrating gene transcription via response elements and thereby affects podocyte integrity [43]. The levels of VitD are mostly lowered in CKD patients due to complex regulatory pathways [44]. These findings emphasize that podocyte structure is closely related to clinical manifestations of kidney injury in our FSGS patient set. Importantly, urinary exosomal miRNAs also correlated with podocyte morphometric and clinical parameters, supporting their role as surrogate markers of podocyte health. Especially miR-181c, miR-3610 and miR-663b showed distinct correlation patterns in FSGS. Our findings regarding miR-181c revealed correlations between expression data and disease markers as well as morphometric parameters, suggesting that this miR is likely to promote pathogenic processes. Conversely, miR-3610 and miR-663b showed opposite patterns of correlation with clinical parameters and podocyte morphometry measurements, therefore allowing assumptions of their beneficial effects. Taken together, our findings introduce a novel approach that, unlike conventional miRNA–mRNA correlation studies, directly integrates urinary exosomal miRNA profiles with high-resolution PEMP-derived podocyte morphometry, highlighting the complementary potential of combining molecular, structural, and clinical readouts in podocytopathies.

Nevertheless, our study has limitations. The small number of healthy controls and the combination of samples from two sequencing cohorts may introduce variability, although batch effects were computationally corrected and no cohort-specific clustering was observed. The CKD cohort in this study included seven etiological subgroups, including diabetic nephropathy (34%), FSGS (18%), and MCD (8%), reflecting typical distributions reported in European CKD biopsy cohorts [45,46]. Nevertheless, the modest sample size limits the generalizability of entity-specific miRNA signatures, and functional studies are required to clarify their role in podocyte biology and CKD pathogenesis. As an exploratory pilot study, our analyses aim to identify biologically relevant associations between urinary exosomal miRNA signatures and podocyte injury, thereby providing a foundation for future mechanistic and longitudinal investigations. Such studies will be essential to determine whether these miRNA patterns change dynamically with treatment or progression, thereby strengthening their utility as predictive biomarkers.

In conclusion, we identified urinary exosomal miRNA signatures that reflect podocyte injury and allow discrimination between CKD entities, with particular diagnostic value for distinguishing FSGS from MCD. Their integration with podocyte morphometry and clinical data offers a multidimensional framework for non-invasive diagnosis, disease monitoring, and mechanistic understanding of podocytopathies, with potential implications for improved treatment of glomerulopathies.

## Figures and Tables

**Table 1:** Number of samples for each diagnosis after filtering. Each diagnosis is differentiated by sequencing site (HGW = University Medicine Greifswald, MD = Otto-von-Guericke University Magdeburg). Total values are shown for each row and column.

Diagnosis	Site HGW	Site MD	Total
Diabetic nephropathy	0	22	22
FSGS	3	9	12
IgA + no blood pressure damage	0	4	4
No blood pressure damage + interstitial nephritis	3	7	10
Minimal Change	0	5	5
ANCA	6	0	6
Hypertensive nephropathy	6	0	6
**Total**	**18**	**47**	**65**

**Table 2:** Patient characteristics of the study population. A total of 65 individuals were included, comprising 62 patients with chronic kidney disease (CKD) and 3 healthy controls. The CKD group encompassed seven etiological subgroups, with diabetic nephropathy, focal segmental glomerulosclerosis (FSGS) being the most common. Shown are age, sex distribution, estimated glomerular filtration rate (eGFR), urinary albumin:creatinine ratio (ACR) and urinary protein:creatinine ratio (PCR), where available. n = number of individuals, n.a. = not applicable.

Characteristics	CKD Patients	Controls
N	65	3
Age: mean (SD)	56 (±14)	73 (±16)
Sex: n female/male (%)	30 (46.2)/35 (53.8)	3 (100)/0
GFR: mean (SD) in mL/min	38.8 (±28.1)	53 (±12.1)
ACR: mean (SD) in mg/g	620.3 (±827.1)	n.a.
PCR: mean (SD) in mg/g	966.6 (±1422.4)	n.a.

## Data Availability

The original contributions presented in this study are included in the article material. Further inquiries can be directed to the corresponding author.
